# Recruitment and Participation of Black Home Health Care Patients in Speech-Based Cognitive Research: Mixed Methods Feasibility Study

**DOI:** 10.2196/87295

**Published:** 2026-05-28

**Authors:** Ian Spens, Zhihong Zhang, Hossein Azadmaleki, Sasha Vergez, Grace Flaherty, Nicole Onorato, James M Noble, Margaret McDonald, Maryam Zolnoori

**Affiliations:** 1 Center for Home Care Policy & Research, VNS Health, New York, NY, United States; 2 Data Science Institute, Columbia University, New York, NY, United States; 3 Columbia University Irving Medical Center, New York, NY, United States; 4 Department of Neurology, Taub Institute for Research on Alzheimer’s Disease and the Aging Brain, GH Sergievsky Center, Columbia University, New York, NY, United States; 5 School of Nursing, Columbia University, 560 W 168th St, New York, NY, 10032, United States, 1 317-515-1950

**Keywords:** recruitment, home health care, cognitive impairment, health disparities, Black patients, feasibility study, speech-based research

## Abstract

**Background:**

Older Black adults remain underrepresented in dementia research, particularly in studies using speech-based methods for early cognitive assessment. Understanding how to effectively recruit and engage this population in research involving audio-recorded interactions is critical to ensuring equitable inclusion and developing culturally responsive study designs. However, recruiting older Black adults into dementia research remains a significant challenge.

**Objective:**

This study assessed the feasibility of recruiting older Black home health care (HHC) patients into speech-based cognitive research and examined the factors influencing participation and participants’ data collection experiences.

**Methods:**

We conducted a convergent mixed methods feasibility study using a 4-component recruitment pipeline at a nonprofit HHC agency: (1) patient identification and study introduction, (2) in-home audio-recorded cognitive assessments, (3) follow-up calls, and (4) audio-recorded patient-clinician encounters. Both patients and their corresponding clinicians were included in this study. Eligible participants were Black adults aged 60 years and older receiving HHC services in New York City. Patient demographic and clinical characteristics were compared between those who consented and those who declined using bivariate analysis. Qualitative feedback was gathered through patient questionnaires and clinician semistructured interviews and was analyzed using reflexive thematic analysis.

**Results:**

Of 246 patients contacted, 71 (28.9%) provided verbal consent and 60 (24.4%) completed cognitive assessments. Five patients were excluded due to health conditions or severe cognitive impairment, leaving 55 eligible participants. Among these participants, retention remained high across study components, including follow-up calls (48/55, 87.3%) and audio-recorded clinician visits (54/55, 98.2%). Patients who did not consent were more likely to have greater complex medical profiles, including higher pain interference (*P*=.01), need for assistance with medication reading (*P*=.04), and polypharmacy (≥5 medications; *P*=.01), while no significant differences were observed for age, gender, or functional status. Qualitative findings demonstrated high acceptability and feasibility of audio recording. Patients reported strong motivation to participate, positive and engaging experiences, comfort with recording, and minimal disruption to care. Clinicians reported ease of integration into workflow, initial discomfort that diminished over time, minor technical challenges, and perceived benefits for communication and patient engagement.

**Conclusions:**

Recruiting Black older adults receiving HHC into speech-based dementia research was feasible and well accepted. Culturally tailored recruitment may enhance equitable participation and guide future research using audio-recorded speech for early cognitive detection.

## Introduction

Mild cognitive impairment (MCI) and early-stage dementia (ED) affect up to 1 in 5 adults aged 60 years and older [1,2], yet more than half of these cases remain undiagnosed, especially in home health care (HHC) settings, where underdiagnosis can surpass 60% [3,4]. Black older adults face a disproportionately high risk, experiencing higher incidence and lower detection of cognitive disorders [5], driven by barriers including restricted access to specialty services, possible biases in clinical evaluations, and limited health literacy [6,7]. Projections indicate that MCI-ED could affect more than 12.7 million individuals by 2050 [8], underscoring the urgency of more equitable, accessible approaches to early detection.

Language impairment often presents early in MCI-ED, positioning speech analysis as a potentially valuable diagnostic tool [9,10]. Speech production is a complex process that engages multiple cognitive domains, including phonetic motor control, working memory, semantic processing, and executive function [11-14], and impairments in these domains can manifest as measurable changes in spoken language. Audio-based cognitive assessment leverages this relationship by extracting acoustic and linguistic features from speech, such as speech rate, pauses, lexical diversity, and semantic coherence, and analyzing them using computational methods (eg, natural language processing and machine learning) to detect patterns of cognitive impairment [15,16]. Compared to traditional assessments, speech-based approaches are noninvasive, low-burden, and scalable. They can be conducted in naturalistic settings, such as HHC visits, enabling ecologically valid and potentially longitudinal monitoring of cognitive changes [15,17]. However, existing speech-processing methods, including automatic speech recognition systems, underperform on the dialects and speech patterns of Black patients [18-20], which is at least in part related to the underrepresentation of a diverse array of voices in training models with respect to age, race-ethnicity, and geography. These disparities underscore the need for more diverse speech corpora that adequately represent Black older adults.

Audio recording of Black patient-clinician verbal communications in HHC encounters provides a unique opportunity to obtain natural speech samples from older adults contending with various health issues associated with aging. Despite its potential, recruiting Black older adults into speech-based dementia research within HHC presents unique challenges. Black older adults remain underrepresented in dementia research, reflecting persistent inequities in participation [21,22]. These disparities are driven by structural barriers (eg, transportation and time burden), interpersonal factors (eg, mistrust of research and health care systems), and study design considerations (eg, perceived relevance and cultural appropriateness) [23-25]. Prior studies also highlight the role of historical discrimination, limited awareness of research opportunities, and lack of culturally tailored engagement strategies in shaping participation [22,24,26]. In HHC settings, additional challenges include privacy concerns related to in-home audio recording, limited familiarity with research participation, competing medical and caregiving demands, and logistical constraints such as variable visit schedules and clinician workload [27-30]. Despite growing recognition of these barriers, limited research has examined the feasibility of recruiting Black older adults receiving HHC into speech-based dementia research.

To address these gaps, this study developed and tested a structured 4-step recruitment pipeline designed to engage older Black adults and their clinicians in speech-based cognitive research within HHC. Specifically, the study focused on the feasibility of recruitment, identified patient-level factors influencing participation, and explored patient and clinician experiences with study enrollment and data collection. The findings are intended to inform culturally responsive and ethically grounded recruitment strategies that can enhance the participation of underrepresented groups in future speech-based dementia studies.

## Methods

### Study Design, Setting, and Participants

This study used a convergent mixed methods design, in which quantitative and qualitative data were collected and analyzed concurrently and integrated during interpretation [31]. This feasibility study is part of a broader research program focused on developing speech-based cognitive assessment methods using audio-recorded patient-clinician interactions and follow-up calls in HHC settings. These approaches leverage speech-derived features to support the early detection of cognitive impairment [17]. This study was conducted at a large nonprofit HHC agency in New York City, which serves about 50,000 patients per year, over 30% of whom are Black. Data collection was conducted across 4 boroughs, including Manhattan, Brooklyn, Queens, and the Bronx, from January 10, 2024, to September 1, 2024. Both HHC patients and clinicians were included in this study.

Eligible patients included are as follows: (1) self-identified Black adults aged 60 years and older who spoke English, were able to communicate independently with clinicians, and had the capacity to provide informed consent; (2) individuals with MCI or ED, defined by an *International Classification of Diseases, 10th Revision* (*ICD-10*) diagnosis (G31.84) or a Clinical Dementia Rating (CDR) score of 0 to 1 and a Montreal Cognitive Assessment (MoCA) score of 18 to 25, with neurologist confirmation; (3) cognitively normal individuals with no documented cognitive impairment, a CDR score of 0, and a MoCA score of ≥26; and (4) participants with sufficient hearing and vision to complete cognitive assessments. Patients were excluded if they had severe cognitive impairment (CDR 2‐3; MoCA 0‐9), major psychiatric or neurological conditions, recent substance abuse, severe communication impairment, unstable or serious medical conditions such as severe pain, active cancer within 2 years, significant sensory impairments, or other conditions limiting participation.

Eligible clinicians were licensed registered nurses, physical therapists, or medical social workers employed by the participating HHC agency, with at least 1 year of HHC experience. All participating clinicians had established clinical relationships with the enrolled patients.

### Ethical Considerations

The study was approved by the institutional review board of the participating agency (approval: E23-01). Information on informed consent, privacy, confidentiality, and compensation is further detailed in the following sections.

### Pipeline for Patient Recruitment and Assessment

#### Pipeline Overview

We used a criterion-based, stratified purposive sampling approach in which participants were intentionally selected based on predefined clinical criteria to ensure the inclusion of both patients with MCI-ED and cognitively normal individuals. This approach aligned with the study’s aim of comparing speech and communication patterns across cognitive status groups to evaluate the feasibility of detecting early cognitive impairment in HHC settings. Patient recruitment and data collection were integrated within routine care and conducted by a trained research assistant (RA) through a 4-step pipeline: (1) identification of eligible patients from electronic health records using predefined inclusion and exclusion criteria, followed by study introduction and verbal consent; (2) in-home cognitive assessments using standardized tools (CDR and MoCA) to confirm eligibility and stratify participants by cognitive status; (3) follow-up phone calls to collect additional speech samples and assess participant experience; and (4) clinician recruitment and audio recording of routine patient-clinician encounters to capture real-world communication. This structured pipeline ensured systematic participant selection, confirmation of eligibility, and consistent data collection aligned with the study objectives.

#### Component 1: Patient Identification and Study Introduction

##### Patient Identification and Outreach

After a HIPAA (Health Insurance Portability and Accountability Act) Waiver of Authorization was approved, we used an R script to identify eligible patients from the HHC organization’s electronic health records based on predefined inclusion and exclusion criteria. An RA then contacted each patient by phone using a standardized script that described (1) the study’s goal of developing a computer program to detect early memory and thinking problems through speech, (2) the patient’s role, (3) the requirement for audio recording, and (4) a US $50 incentive upon assessment completion. If a caregiver answered the phone, the RA explained the study and then requested to speak directly with the patient to confirm interest and obtain verbal informed consent.

##### Strategies to Build Trust and Comfort

To improve patients’ experience with participation, the RA received comprehensive training in interpersonal and linguistic strategies. For instance, the RA periodically paused during conversations to allow patients to process information and respond. They also substituted clinical terms with everyday language, such as referring to “questionnaires” instead of “cognitive assessment tests” and “memory and thinking problems” rather than “MCI-ED.” By validating patients’ comments or concerns (eg, “That’s a good point” or “I understand why you might feel that way”) and emphasizing the study’s respect for participants’ well-being, the RA fostered a sense of trust and comfort.

### Component 2: In-Home Cognitive Assessments With Audio Recording

#### Participant Scheduling and Informed Consent

After obtaining verbal consent and scheduling an appointment in component 1, the RA visited the patient’s home to administer the cognitive tests. The RA confirmed each appointment in advance to address potential scheduling conflicts (eg, changes in the patient’s health). Upon arrival at the patient’s home, the RA provided a brief overview of the study, answered questions, completed the informed consent process, and obtained written informed consent. When caregivers were present, the RA invited them to observe the consent process but asked them not to influence the patient’s responses during the cognitive assessments.

#### Audio Recording Cognitive Assessments

The RA administered 2 standardized cognitive assessments (CDR and MoCA) [32,33]. The CDR evaluates 6 domains, including memory, orientation, judgment or problem-solving, community affairs, home or hobbies, and personal care. The CDR provides a composite score ranging from 0 (no dementia) to 3 (severe dementia) [33]. The MoCA assesses memory, attention, language, visuospatial ability, executive function, and orientation, with a total score ranging from 0 to 30 [32]. The RA received formal training in both assessments through the University Alzheimer’s Center, certification from Washington University, and additional orientation at the HHC organization.

Cognitive assessments were audio-recorded using the Saramonic Blink500 Pro B2 system, which consists of 2 wireless transmitters paired with Countryman DK6A headset microphones. Audio signals were transmitted to a receiver compatible with iPhone devices, ensuring high-quality, synchronized recordings. An eighth-generation iPad served as a backup recording device to support quality control. All recordings were securely uploaded to an encrypted server accessible only to the research team. The RA did not provide clinical feedback based on assessment results; however, patients with low cognitive scores were advised to consult their health care providers.

#### Strategies to Enhance Test Accuracy and Patient Comfort

To enhance test accuracy and patient comfort, the RA employed several strategies: (1) engaged patients in brief conversation before and during assessments to alleviate anxiety and foster a supportive environment; (2) encouraged patients who appeared uncertain to provide their best possible responses without concern for perfection; (3) offered breaks when patients showed signs of fatigue to reduce stress and maintain data integrity; (4) politely asked caregivers to refrain from assisting during the assessments to preserve the validity of responses; and (5) requested a reduction in background noise, such as lowering the television volume, to improve audio-recording quality while respecting patient preferences if they declined. Clear audio was essential for capturing high-quality speech data.

### Component 3: Follow-Up Calls Recording

#### Timing and Rationale

Follow-up phone calls were conducted approximately 3 to 5 weeks after each patient’s in-home cognitive assessment. The primary goals of these calls were to (1) collect additional speech samples in a different conversational setting, (2) assess patients’ recall of the initial visit, and (3) gather feedback on their experience with being audio-recorded. Although the short interval was not intended to capture major changes in cognitive status, it allowed for the observation of minor fluctuations or day-to-day variations that may influence speech characteristics. It also provided patients with enough time to reflect on their overall experience with audio recording. In line with the study’s feasibility focus, these follow-up conversations offered valuable insights into participants’ willingness to complete multiple recordings and the potential adaptability of speech-based assessments across varying contexts.

#### Follow-Up Calls Recording

Follow-up phone calls were conducted by the same RA who administered the in-home assessments, ensuring continuity and rapport. Each call lasted approximately 15 minutes and was audio-recorded using the Zoom mobile app’s recording feature. The RA referenced details from the initial visit to encourage patient engagement and trust, following a standardized guide that prompted discussions of recent life experiences, perceived health changes, and impressions of study procedures. Immediately after each call, audio recordings were labeled with unique participant identifiers and uploaded to a secure, access-restricted server. All data handling conformed to institutional and federal privacy regulations.

### Component 4: Clinician Recruitment and Audio Recording of Patient-Clinician Encounters

#### Clinician Recruitment and Consent

Clinicians were recruited through multiple outreach methods, such as mass emails, direct text messages, presentations about the importance of the study at weekly meetings, and advertisements on the HHC organization’s internal social media. Interested clinicians attended a 15-minute orientation (in person or virtual) covering the following: (1) a study overview, (2) their role in audio recording HHC encounters, (3) privacy measures, and (4) compensation of US $80 for recording 2 encounters. Written consent was obtained from clinicians in person or finalized after virtual meetings. The RA then contacted patients who had completed cognitive assessments to request consent for audio recording their HHC encounters, informing them that their clinicians had enrolled.

#### Audio Recording of Patient-Clinician Communication

Clinicians were alerted when they had a patient on their caseload who had consented to the audio recording of their clinical visit. Clinicians then completed the recording during their next routine HHC encounter. During each audio-recorded encounter, clinicians ensured the patient’s comfort with being recorded and asked patients or caregivers to minimize background noise (eg, lowering the TV volume). After each encounter, recordings were uploaded to a secure internal server. This process yielded spontaneous, real-world patient-clinician communication, enriching the dataset of speech patterns relevant to the early detection of cognitive impairment.

### Data Collection

#### Quantitative Data Collection

##### Patient Recruitment Data

To assess recruitment and retention feasibility, we tracked the number of Black HHC patients who consented, declined, or withdrew at each study stage, documenting reasons for both participation and nonparticipation (eg, privacy concerns, scheduling conflicts) whenever available.

##### Factors Associated With Patient Participation

To explore factors influencing consent decisions, we collected demographic (eg, age, gender) and clinical (eg, comorbidities, physical functioning) data from the Outcome and Assessment Information Set (OASIS), a standardized dataset mandated by the Centers for Medicare & Medicaid Services [34]. This dataset provides patient-level details such as medical conditions, living arrangements, and support systems.

### Qualitative Data Collection

To assess patient and clinician experiences with the study procedures, we collected qualitative data through structured follow-up interviews and open-ended questionnaires.

#### Patient Feedback

During follow-up calls conducted 3 to 5 weeks after the in-home cognitive assessments (see component 3), patients were asked about their experiences with the recruitment process, completing cognitive tests at home, being audio-recorded during the visit, and their comfort with the use of speech-based technology to detect early signs of cognitive decline. They were also invited to share concerns about privacy and their overall impressions of the study. These phone interviews were audio-recorded and conducted by the trained RA using a standardized interview guide, which is provided in Multimedia Appendix 1.

#### Clinician Feedback

Clinician feedback was collected by the same trained RA using a semistructured approach across 3 modalities: Zoom interviews, phone calls, and open-ended questionnaires, based on clinician availability. A standardized interview guide was used to ensure consistency across data collection methods and is provided in Multimedia Appendix 1. The guide included open-ended questions assessing clinicians’ experiences with audio-recording patient encounters, including (1) overall experience with recording, (2) usability of the recording device, (3) integration of recording into the HHC workflow and associated challenges, (4) perceived impact of recording on communication with patients and caregivers, (5) perceived impact on patient outcomes, and (6) additional recommendations or comments. This approach allowed for flexible yet systematic collection of clinician perspectives on the feasibility, acceptability, and practical implementation of audio recording in routine care.

### Data Analysis

#### Quantitative Data Analysis

We used descriptive statistics (eg, frequency distributions) to characterize the sample, followed by bivariate analysis to compare characteristics between consenting and nonconsenting patients. All continuous variables, such as age and daily functioning scores, were converted into categorical or ordinal groups to facilitate appropriate statistical testing based on the distribution of the data. Accordingly, the *χ*² test was used for categorical variables (eg, gender), and the Cochran-Armitage [35] test for trend was applied to ordinal variables (eg, level of daily functioning) to detect monotonic trends across ordered groups. All analyses were conducted in Python using SciPy [36]. Statistical significance was set at *P*<.10, given the exploratory nature of this feasibility study and the relatively small sample size, to allow for the identification of potential trends that may inform future research.

#### Qualitative Data Analysis

Interview recordings were transcribed using Amazon Web Services Transcribe and reviewed by the RA for accuracy [37]. All transcripts were deidentified and securely stored. Patient and clinician interviews were analyzed separately using reflexive thematic analysis following the 6-phase approach by Braun and Clarke [38]: (1) Familiarization: 2 researchers independently reviewed transcripts and documented preliminary observations; (2) Initial coding: meaningful units were inductively coded using a structured Microsoft Excel workbook shared through OneDrive, which served as a dynamic codebook; (3) Theme development: related codes were grouped into preliminary themes reflecting patterns across the data; (4) Reviewing themes: the research team collaboratively refined themes for internal coherence and alignment with study aims; (5) Defining and naming themes: final themes were clearly defined to capture their central meanings; and (6) Reporting: representative deidentified quotations were selected to illustrate themes related to recruitment, retention, audio-recording feasibility, and participant experiences. Throughout the analytic process, the research team engaged in iterative discussions to reflect on coding decisions, resolve discrepancies, and refine theme development. Reflexivity was maintained by acknowledging the researchers’ roles in data collection and analysis and by critically considering how their perspectives and assumptions may have influenced interpretation.

To enhance qualitative rigor, we applied strategies aligned with trustworthiness criteria. Credibility was supported through the use of a standardized semistructured guide, triangulation of data sources (patients and clinicians), and inclusion of representative quotations to ground findings in participants’ perspectives. Dependability was ensured through a systematic and transparent analytic process, including the use of a shared codebook (Multimedia Appendix 2) and documentation of analytic decisions across team meetings. Confirmability was strengthened by maintaining an audit trail of coding and theme development decisions and through team-based review to minimize individual bias. Transferability was supported by providing detailed descriptions of the study setting, participant characteristics, and recruitment context to enable assessment of applicability to similar HHC populations.

## Results

### Overview of Patient Recruitment and Participation Across the Study Pipeline

**Figure 1.**
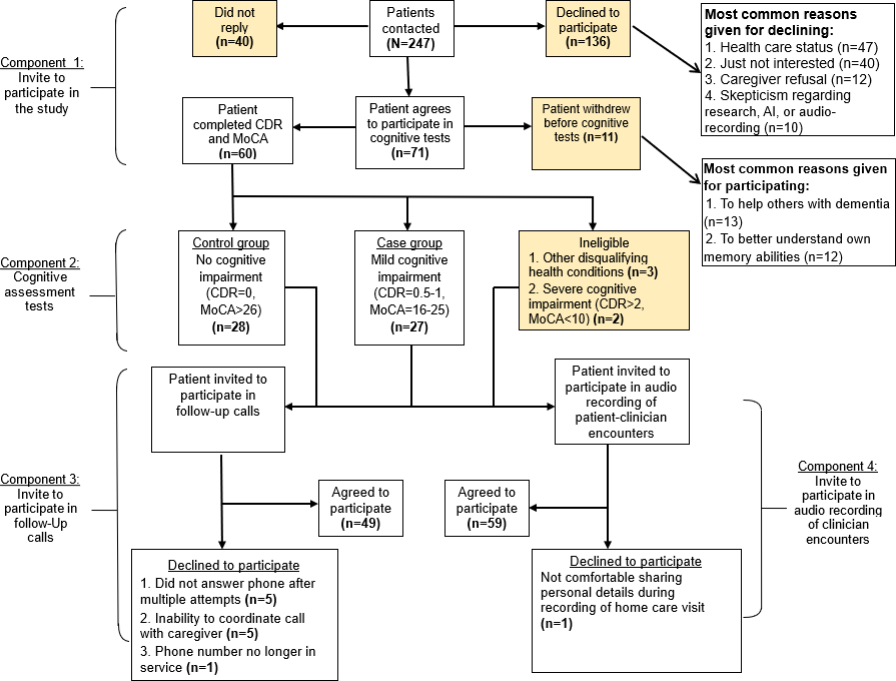
Patient recruitment and participation across the study pipeline. AI: artificial intelligence; CDR: Clinical Dementia Rating; MoCA: Montreal Cognitive Assessment; RA: research assistant.

Figure 1 outlines the recruitment and participation flow across the 4 components of the study. In component 1, we attempted contact with 246 patients by phone. Of these, we were unable to reach 40, and 135 declined participation due to reasons such as health issues (47/135, 35%), lack of interest (40/135, 30%), caregiver refusal (12/135, 9%), or skepticism about research, artificial intelligence, or audio recording (10/135, 7%). The reasons listed represent the most common reasons identified and are not intended to be an exhaustive list. Ultimately, 71 patients verbally consented to participate. Before cognitive testing in component 2, 11 withdrew, leaving 60 who provided written consent and completed the assessments. Of these, 5 were deemed ineligible due to disqualifying health conditions (n=3) or severe cognitive impairment (n=2) based on the screening results. All 55 eligible patients were invited to participate in component 3 (follow-up phone calls); 48 (87%) agreed, while 7 (13%) declined due to unreturned calls (n=4), caregiver coordination issues (n=2), or a disconnected phone line (n=1). In component 4, 54 of 55 (98%) eligible patients consented to audio record their HHC visits, with 1 declining due to discomfort sharing personal details. Overall, the recruitment process yielded a consent rate of 24% (60/246) from initial outreach and strong participation rates across cognitive assessment (60/71, 85%), follow-up calls (48/55, 87%), and audio-recorded visits (54/55, 98%), demonstrating the feasibility of engaging Black patients in audio-based research within HHC settings. Ten participating clinicians provided feedback, including 5 registered nurses, 4 physical therapists, and 1 medical social worker. Participants included 2 Black, 5 White, 2 Hispanic, and 1 Asian clinician. Most (7/10, 70%) reported 1 to 5 years of experience, while 3 (30%) reported more than 5 years.

### Factors Associated With Patient Consent Decisions

Table 1 compares the characteristics of those who provided written consent (60/71, 84.5%) to those who did not (146/206, 71%), a group that includes both initial decliners and patients who withdrew prior to written consent. No significant differences were observed in gender, age, insurance type, living situation, social isolation, anxiety levels, or activity of daily living scores. However, nonconsenting patients were more likely to require assistance reading medication labels (*P*=.04), experience frequent pain interference (*P*=.01), take 5 or more medications (*P*=.01), and have a diagnosis of heart disease (*P*=.05). They also reported marginally higher levels of exhaustion (*P*=.08). These findings suggest that greater medical burden and functional limitations may hinder research participation among HHC Black patients.

**Table 1.** Characteristics of patients who consented to or declined study participation.

Classification	Not consented (n=146), n (%)	Consented (n=60), n (%)	*P* value
Female	98 (67.1)	41 (68.3)	>.99
Age, y			.19
60‐69	37 (25.3)	26 (43.3)	
70‐79	56 (38.4)	22 (36.7)	
80‐89	39 (26.7)	22 (36.7)	
≥90	14 (9.6)	3 (5)	
Insurance			.27
Dually eligible Medicaid or Medicare	28 (19.2)	16 (26.7)	
Medicaid	12 (8.2)	3 (5)	
Medicare	86 (58.9)	35 (58.3)	
Private insurance	20 (13.7)	5 (8.3)	
No insurance	0 (0)	1 (1.7)	
Help needed for medication reading (literacy)			.04^a^
Never or minimal assistance needed	94 (64.4)	49 (81.7)	
Sometimes	39 (26.7)	10 (16.7)	
Always	12 (8.2)	1 (1.7)	
Living situation			.82
Alone	59 (40.4)	26 (43.3)	
With others	87 (59.6)	34 (56.7)	
Social isolation			.11
Never	108 (74)	49 (81.7)	
Rarely	18 (12.3)	9 (15)	
Sometimes	19 (13)	2 (3.3)	
Reports exhaustion	62 (42.5)	17 (28.3)	.08^b^
Shortness of breath			.75
None	37 (25.3)	16 (26.7)	
With major exertion	44 (30.1)	20 (33.3)	
With moderate exertion	56 (38.4)	23 (38.3)	
With minimal exertion	7 (4.8)	1 (1.7)	
Frequency of anxiety			.62
Never	58 (39.7)	25 (41.7)	
Occasionally	51 (34.9)	17 (28.3)	
Frequently	37 (25.3)	18 (30)	
ADL^c^ score			.75
Independent or minimal assistance needed	1 (0.7)	1 (1.7)	
Little assistance needed	23 (15.8)	8 (13.3)	
Some assistance needed	104 (71.2)	41 (68.3)	
Completely dependent	18 (12.3)	10 (16.7)	
Pain interference with daily life			.01^a^
Rarely	16 (11)	16 (26.7)	
Occasionally	65 (44.5)	21 (35)	
Frequently	36 (24.7)	12 (20)	
Almost constantly	6 (4.1)	0 (0)	
Taking 5 or more medications	136 (93.2)	48 (80)	.01^a^
Urinary incontinence	61 (41.8)	24 (40)	.94
Heart disease	20 (13.7)	2 (3.3)	.05^a^
Diabetes	59 (40.4)	22 (36.7)	.73

a *P*<.05.

b *P*<.10.

c ADL: activity of daily living.

### Participants’ Experience With Audio-Recording Pipeline

#### Patients’ Perspectives

##### Theme 1: High Acceptability and Motivation to Participate

Patients were highly receptive to participating in the study, with many describing their decision to enroll as immediate and meaningful. A common motivation was contributing to research on cognitive impairment, as reflected in statements such as “Anything I can do to help people with dementia.” Others expressed interest in understanding their own cognitive health (eg, “It’d be nice to see how I do on these”). While a small number of patients initially declined participation due to competing demands or health concerns, some later agreed following additional outreach, suggesting openness when engagement was sustained.

##### Theme 2: Generally Positive and Engaging Experience

Most patients reported that the cognitive assessments were enjoyable and stimulating. Participants described the experience as “mentally refreshing” and appreciated the interactive nature of the tasks. Although a minority reported neutral experiences or difficulty recalling their impressions, no participants described the assessments as burdensome.

##### Theme 3: Comfort With Audio Recording

Patients consistently reported comfort with audio recording during both cognitive assessments and HHC visits. Many described the recording devices as unobtrusive, noting that they “forgot [they were] even recording” or “didn’t notice it was there.” A small number of participants expressed initial concerns, such as privacy or device preference, but these concerns were resolved after clarification of study procedures.

##### Theme 4: Minimal Disruption to Care

Patients reported that audio recording did not interfere with their care experience. Recorded visits were described as comparable to routine encounters, with participants noting that interactions with clinicians felt natural and unchanged (eg, “It felt like any other nurse visit”). No participants reported negative impacts on their care due to recording.

### Clinicians’ Perspectives

#### Theme 1: High Acceptability and Ease of Integration

Clinicians consistently described audio recording as easy to implement and well integrated into routine care. Many reported that the process was “straightforward” and “did not interfere at all” with workflow, with some noting that they “started as soon as [they] opened the chart and went through the visit as usual.” Several clinicians also reported that they would “forget that [they were] recording,” indicating minimal burden during routine visits.

#### Theme 2: Initial Discomfort That Diminishes Over Time

Some clinicians described an initial sense of awkwardness when being recorded, particularly during early encounters. For example, one clinician noted feeling “a little awkward” during the first few visits. However, this discomfort typically diminished with experience, and subsequent encounters were described as natural and routine. A few clinicians also noted that both they and their patients became more self-conscious when aware of the recording.

#### Theme 3: Minor Technical and Workflow Challenges

Although overall usability was high, some clinicians reported minor technical challenges, including microphone setup difficulties and interruptions due to phone notifications. One clinician noted that “the microphone was somewhat difficult to set up and caused some stress,” while others described recordings stopping unexpectedly. Several clinicians adapted by using their phones instead of the microphone, which they found more reliable and easier to integrate into their workflow. Additional challenges included coordinating recordings within time-constrained visits and enrolling patients before the end of care episodes.

#### Theme 4: Minimal Impact on Communication With Some Contextual Considerations

Most clinicians reported that audio recording did not substantially affect communication with patients, noting that interactions remained consistent with usual care (eg, “I carry out my visits the way I would with or without the recording”). However, some clinicians suggested that patients might be less willing to discuss sensitive topics while being recorded. In isolated cases, clinicians also reported increased self-consciousness among patients.

#### Theme 5: Perceived Benefits for Communication and Patient Engagement

Many clinicians perceived potential benefits of audio recording for improving care delivery. Some reported increased self-awareness, noting that recording encouraged them to “slow down” and “pay extra attention to detail.” Others described making additional efforts to engage patients in conversation, and some observed increased patient participation during recorded visits. Clinicians also noted that recordings could support better communication and information management, with one stating that “recording the session could help manage [clinical] information more easily.” However, several clinicians noted that time constraints may limit the routine use of recordings in practice.

### Integration of Quantitative and Qualitative Findings

Integration of quantitative and qualitative findings showed that lower consent rates among patients with greater medical burdens were consistent with reported barriers such as pain and competing health demands. In contrast, high participation across study components aligned with patients’ motivation to contribute to research and positive study experiences. Both patients and clinicians reported that audio recording was acceptable and minimally disruptive, supporting the feasibility of integrating speech-based data collection into routine HHC (Table 2).

**Table 2.** Joint display of quantitative and qualitative findings on recruitment, participation, and feasibility.

Quantitative findings	Qualitative findings	Integrated interpretation
Low initial consent (60/246, 24%); high assessment completion among those who agreed (60/71, 85%)	Patients who enrolled often described participation as an easy decision and were motivated by contributing to dementia research and understanding their own memory. Some initially declined but agreed after follow-up.	Initial recruitment was challenging, but willingness increased when patients perceived personal relevance and altruistic value, suggesting the importance of engagement and follow-up.
Lower consent among patients with higher medical burden	Some patients declined or delayed participation due to pain, competing health demands, or time constraints.	Greater health and functional burden may act as barriers to research participation in home health care populations.
High participation in follow-up calls (48/55, 87%) and audio-recorded visits (54/55, 98%)	Patients reported positive experiences, found cognitive testing engaging, and remained willing to participate in multiple study activities.	Once enrolled, patients demonstrated strong retention and engagement, supporting the feasibility of the multistep study pipeline.
High acceptance of audio recording	Patients described the recording devices as comfortable and unobtrusive, with minimal concerns (eg, initial privacy concerns or device preference). Clinicians similarly reported that recording was generally easy to implement.	Audio recording was highly acceptable and feasible for both patients and clinicians, with only minor and manageable concerns.
High completion of audio-recorded visits (54/55, 98%)	Patients reported that recorded visits felt similar to routine care and did not disrupt interactions. Clinicians noted minimal workflow disruption, with only occasional technical issues or initial adjustment.	Audio recording can be integrated into routine home health care with minimal disruption to care delivery.

## Discussion

### Principal Findings

This study examines the recruitment and participation process for an audio-recorded speech study in an HHC setting. Of the patients initially contacted, approximately 1 in 4 agreed to participate, demonstrating a moderate recruitment rate among an older adult population, which is particularly notable given our exclusive focus on older Black patients receiving HHC, a population often underrepresented in cognitive impairment research. Importantly, we found that patients with low literacy and greater medical complexity (eg, polypharmacy, frequent pain interference, and heart disease) were more likely to decline participation, highlighting the need for research strategies to account for or identify better ways to include individuals with a greater burden of chronic conditions. Despite these challenges, both clinicians and patients who participated reported positive attitudes toward audio recording, with minimal disruption to standard care. This underscores the potential for innovative, noninvasive diagnostic methods, such as speech-based assessments, to be integrated into routine HHC visits for earlier detection of MCI-ED.

Recruiting older adults, particularly those with MCI-ED, presents unique challenges and often leads to lower participation rates in research [39,40]. Numerous studies have reported the underrepresentation of older adults in clinical trials, especially among those in the oldest age groups [41,42]. In contrast, our study achieved relatively balanced enrollment across age groups, suggesting that targeted outreach and tailored messaging can enhance the inclusion of older adults at varying levels of cognitive and medical complexity. Nevertheless, although our overall consent rate was slightly lower than the 38% reported by other studies that focused on dementia research [39], it remains noteworthy in the context of reaching Black patients with multiple comorbidities [43,44].

Older adults with MCI-ED often face multiple barriers to research participation, such as lack of interest, caregiver discouragement, or feeling overwhelmed by their conditions. A systematic review on the recruitment and retention of older adults in clinical research found that lack of interest and external discouragement were among the most common reasons for refusal [45]. Consistent with these findings, our study also identified these 2 barriers. Additionally, our findings demonstrated that patients with greater medical complexity (eg, polypharmacy and frequent pain interference) were more likely to decline, consistent with research showing that poor health status can impede enrollment [46,47]. Several factors may explain these differences. Patients with higher symptom burden and functional limitations may experience fatigue, pain, or competing care demands, reducing their capacity or willingness to engage in research activities. In addition, managing multiple medications and chronic conditions may increase cognitive and logistical burdens, making participation in additional study procedures less appealing.

Importantly, integrating qualitative findings provides additional insight into these patterns. While patients with greater medical complexity were less likely to participate, those who enrolled were often motivated by altruism and a desire to understand their cognitive health. Qualitative data also suggest that some barriers, such as pain or time constraints, may be modifiable. For example, several patients who initially declined audio recording during home visits subsequently expressed interest in participating following follow-up contact, suggesting that recruitment decisions may be dynamic rather than fixed. In addition, participants consistently reported that audio recording of patient-clinician encounters was unobtrusive and did not interfere with care. Together, these findings suggest that although greater disease burden may limit initial participation, targeted strategies, such as leaving contact information and inviting patients to reach out if they later express interest, along with clear communication and reassurance about study procedures, may improve recruitment among higher-risk individuals.

While quantitative findings indicated that patients with greater medical burden (eg, polypharmacy and pain interference) were more likely to decline participation, qualitative data provide important context for these patterns. Many participants reported that their decision to enroll was driven by altruistic motivations (eg, contributing to dementia research) and personal interest in understanding their cognitive health. Notably, qualitative findings also suggest that initial barriers such as pain or time constraints may be modifiable; for example, some patients who initially declined later agreed to participate following follow-up contact. In addition, participants who enrolled generally reported positive experiences and minimal burden, particularly regarding audio recording, which was perceived as unobtrusive. Together, these findings suggest that while higher medical burden may limit initial participation, targeted strategies such as follow-up engagement, clear communication, and reassurance about study procedures may help overcome initial reluctance and improve recruitment. A key outcome of this study was the generally positive reception of audio recording by both patients and nurses, which supported high participation rates in the latter components (eg, audio-recorded home visits). Previous work suggests that embedding research procedures into existing care routines, rather than adding separate appointments, enhances participant engagement and retention [48,49]. In our study, face-to-face interactions during home visits likely fostered trust and comfort, aligning with research showing that relational continuity in HHC can improve study adherence [49,50]. Audio recording was perceived as unobtrusive and straightforward to integrate into routine workflows, affirming earlier findings on the feasibility of speech-based data collection in HHC settings [30].

### Implications for Future Research

Future research should prioritize the development of tailored recruitment strategies that address the diverse needs and health complexities of older adults with MCI-ED, particularly those with severe medical conditions who are often underrepresented in research. Customized approaches that take into account both medical and personal circumstances such as personalized communication, flexible participation options, and targeted outreach can overcome specific barriers. Additionally, clear and accessible communication materials that explain the study’s purpose, benefits, and safety measures can help build trust, reduce skepticism, and minimize caregiver refusals. Such approaches could reduce both logistical and psychological barriers, ultimately improving the diversity and representativeness of study samples.

The application of audio recordings for speech analysis has shown significant promise as a tool for the early detection of MCI-ED [16,51]. The positive feedback from study participants highlights the feasibility and potential for integrating this method into routine HHC diagnostics. Future research should further investigate and validate the effectiveness of audio-recorded speech data in supporting the early identification of MCI-ED. By enabling early detection and timely interventions, this approach could potentially slow or even halt the progression of cognitive decline in this vulnerable population, thereby improving patient outcomes and quality of life.

### Limitations

Despite offering valuable insights, our study faces several limitations that warrant consideration. First, a substantial number of patients declined participation during recruitment, which may have introduced selection bias and limited the generalizability of the findings, particularly among individuals with more intensive health needs who may be underrepresented. As a result, the sample may not fully capture the experiences of the most severely affected segments of the MCI-ED population, who are often the primary targets for early diagnostic tools and interventions. However, given that this step was intended to assess the feasibility of the approach, it reflects real-world engagement with an understudied population. Second, we did not collect detailed demographic information for clinicians and only captured limited characteristics (eg, professional role and years of experience), which limits the interpretation of clinician perspectives. Third, because the study was conducted within a technology-supported environment, findings may not be transferable to settings with limited access to technology or among populations more skeptical of artificial intelligence-based research. Fourth, although the study by design specifically focused on including older Black patients, barriers faced by other racial and ethnic groups in this setting could not be determined. Nonetheless, the approach taken in this study will inform subsequent steps, including engaging more groups of older adults in New York and elsewhere. Finally, while most participants were comfortable with audio recording, some expressed privacy concerns that may have led to hesitation in sharing sensitive information and could have impacted the completeness and accuracy of the data. This finding highlights the need for effective communication and trust-building with research teams, as well as the importance of effective data security measures in these and other platforms.

### Conclusion

This study demonstrates the feasibility and acceptability of an audio-recording pipeline for speech-based cognitive assessment among older Black adults in HHC settings. Although recruitment was hindered by high medical complexity among nonparticipants, those who enrolled reported minimal disruption from audio recording and expressed positive attitudes toward the research. These results highlight the promise of speech-based tools for early MCI-ED detection and underscore the importance of inclusive recruitment strategies and technology safeguards, particularly for medically vulnerable populations.

## Supplementary material

10.2196/87295 Multimedia Appendix 1 Interview guide.

10.2196/87295 Multimedia Appendix 2 Codebook.

## Abbreviations

CDR: Clinical Dementia Rating
ED: early-stage dementia
HHC: home health care
HIPAA: Health Insurance Portability and Accountability Act
*ICD-10*: *International Classification of Diseases, 10th Revision*
MCI: mild cognitive impairment
MoCA: Montreal Cognitive Assessment
OASIS: Outcome and Assessment Information Set
RA: research assistant
