# Supplementary material for: Recruitment and Participation of Black Home Health Care Patients in Speech-Based Cognitive Research: Mixed Methods Feasibility Study
Source: JMIR Form Res. 2026 May 28;10:e87295. doi: 10.2196/87295 (PMC13218563; doi:10.2196/87295)
Supplement: Multimedia Appendix 1 [file formative-v10-e87295-s001.docx]

**Appendix 1 Interview Guide**

**Patients’ follow-up questions**

1. Overall, how would you describe your experience participating in the study, including the in-home assessment and being audio-recorded?
2. What concerns, if any, do you have about privacy or the use of speech-based technology for detecting early cognitive changes?

**Clinician interview questions**

1. Please describe your overall experience while audio-recording your patient encounter(s).
2. How well did the recording process fit into your homecare workflow? What challenges, if any, did you experience?
3. How does the fact that an encounter is being audio-recorded affect your communication with patients and their caregivers?
4. What do you think is the overall impact of audio-recording encounters on patients’ outcomes?
5. (Optional) Please feel free to share any recommendations you have as to how we can improve the audio-recording process to make it easier and more comfortable for you and your patients.
