# Supplementary material for: Recruitment and Participation of Black Home Health Care Patients in Speech-Based Cognitive Research: Mixed Methods Feasibility Study
Source: JMIR Form Res. 2026 May 28;10:e87295. doi: 10.2196/87295 (PMC13218563; doi:10.2196/87295)
Supplement: Multimedia Appendix 2 [file formative-v10-e87295-s002.docx]

**Appendix 2 Codebook**

1. **Codebook for Patient Follow-up Phone Call Interviews**
   The codebook for patient follow-up phone call interviews was not made available due to the presence of protected patient information in some quotations.
2. **Codebook for Clinician Interviews**
   See below table.

| **Clinician** | **Contact Method** | **Q1.** Please describe your overall experience while audio-recording your patient encounter(s). | **Q1.5**. Device Usability | **Q2.** How well did the recording process fit into your homecare workflow? What challenges, if any, did you experience? | **Q3.** How does the fact that an encounter is being audio-recorded affect communication with patients and caregivers? | **Q4.** What do you think is the overall impact of audio-recording encounters on patients’ outcomes? | **Q5.** Additional recommendations (optional) |
| --- | --- | --- | --- | --- | --- | --- | --- |
| RN1 | Phone Call | No issues, really easy to set up mic, patient had ease doing it to due to previous experience.  *Positive experience.* | *Easy* | Really easy, started as soon as opened chart, went through visit as usual; didn't challenge workflow. Most challenging part was getting file to me in future.  *No issues* | For me it doesn't affect me; I have experience recording in the past. I'm used ot having a mic on me. Didn't change behavior; minor at most. Possible issue with other patients though.  *No effects observed.* | Probably wouldn't use recording/listen after. With infinite time it would be very useful, would help verify certain details about visit. However, time constraints make it difficult to do this regularly. Some other clinicians make brief audio-recordings summarizing the visit.  *Help patients receive proper care.* | Nothing to improve on for me. However, nurses without background in audio-recording may need additional training.  *Time constraints limit participation*  (Separate convo) |
| PT1 | Survey Entry | The process is easy. In my experience there are 2 challenges that I try to address- to keep the recording process uninterrupted when Pt needs to do mobility activities like stair climbing tng or outdoor activities- I need to ensure that the recording is continuing; 2nd is how to set up the patient as quickly as I could on each session to capture as much interaction I have with the patient  *Positive experience.* | *Easy*  *Interruptions* | It did not interfere at all in my workflow. It just added a few extra steps but nothing significant. As to challenges, I have mentioned it in my answer in the previous question  *No issues* | None at all. I carry out my visits the way I would with or without the recording.  *No effects observed.* | I can’t gauge the impact of audio recording patient encounters on patient’s outcomes at this time. However on the sessions I did recordings, patients tends to demonstrate increased participation in the PT sessions.  *Increases patient participation.* | I could not think of any recommendations at this time. I think the set up you have established is pretty efficient. The challenge on keeping the recording continuous will always be present depending on signal strength and planned activities for the session. |
| RN2 | Survey Entry | It was overall very smooth; everything was set up through email and then the researcher Ian set me up with the information I needed to do everything, then they promptly followed up with submitting the recordings.  *Positive experience.*  *Difficulty recording patients before end of episode.* | *Easy* | The main challenge is the patients by the time they are deemed eligible for the study, or consent, they may no longer be on treatment or perhaps with another discipline besides nursing so cannot record a visit.  *No issues* | You feel a little awkward and nervous that you don’t want to sound silly, but it forces you to slow down.  *“Awkward” – clinician end*  *Increased self-awareness* | Can be useful to study what communication is best for delivering healthcare  *Improve communication of information.* | Thank you!  *Time constraints limit participation* |
| RN3 | Zoom Call | Good overall. Felt a little awkward during the first 1 or 2 visits, but felt comfortable and natural during subsequent recordings.  Really enjoys participating in the study (R01), and is hopeful that the algorithm will help keep patients out of the hospital.  *Positive experience.* | *Challenging* | The microphone was somewhat difficult to set up and caused some stress. Found that recording was much easier with iphone mic and yielded similar quality.  *Challenge with microphone setup. Eliminated by using iPhone mic.* | At first conversation felt somewhat awkward due to being aware of being recorded, but that went away after the first 2 visits. He reported that the recording may actually encourage him to pay extra attention to detail.  *“Awkward” – clinician end*  *Increased self-awareness* | “It’s good to know what goes on during visits.”  Helps provide a clear picture of pts’ experience during the home care visit, allows clinicians to review things they may have otherwise missed.  Recording clinicians also encourages them to do the best they can.  *Improve communication of information.*  *Help patients receive proper care.* | Prefers not to use microphone (see Q2). |
| RN4 | Zoom | Good experience overall. Patients were all interested in participating.  However, time constraints made it difficult for patients to participate.  *Positive experience.*  *Difficulty recording patients before end of episode.* | *Easy* | Fits smoothly into clinical workflow; would confirm that she had begun recording, and visit went as normal.  *No issues* | Reports making additional effort to encourage patients in more dialogue to provide as much speech data as possible.  *Increases clinician engagement with patients* | There’s a lot of information when you open up the case; recording the session could definitely help manage that information more easily.  *Improve communication of information.* | Clarifying more details for follow-up with patients.  *Time constraints limit participation* |
| PT2 | Zoom | Good experience overall, patients enjoy participating as well.  *Positive experience.* | *Easy*  *Interruptions* | Very easily; forgets that he’s recording the visit.  However, the recording will cut out if you get an email or a call, and requires resuming the recording.  *No issues* | No effect at all; patient also forgets that visit is being recorded. Feels just like a normal visit.  *No effects observed.* | Feels the study (R01) has a lot of potential to help patients. However, doesn’t see self using recordings for own use.  *Help patients receive proper care.* | More advanced notice when a patient is recruited. |
| PT3 | Zoom | Straightforward, setup of mic is easy. Very helpful that pts are informed by Grace about full details of study beforehand.  *Positive experience.* | *Easy* | Impact is minimal; no issues at all fitting into workflow.  *No issues* | Dialogue was pretty natural.  In one case a patient found a bedbug in their apartment, wanted to stop recording.  *May limit communication of sensitive info.* | Sees the value in this research in getting patients appropriate care.  Clinicians using recordings to review their visits could help increase their self-awareness. However, she doesn’t see herself doing that personally because of a lack of time.  *Help patients receive proper care.*  *Increase clinician’s self-awareness.* | Patients will sometimes assume that Michelle is more involved with the study than she is. (E.g. One patient thought their PT would record remaining visits, but PT wasn’t participating. Pts will also ask questions that Michelle doesn’t know the answer to.” |
| MSW1 | Forms | My experience was fairly straightforward. The recording device wasn't working so I just recorded the visit on my VNS phone, which seemed to work well.  *Positive experience.* | *Difficult* | The device and microphone didn't work but I switched to my VNS phone rather seamlessly.  *Challenge with microphone setup. Eliminated by using iPhone mic.* | My communication remained the same. I went through all of my assessment questions the same as I do with all of my patients.  *No effects observed.* | Hopefully it was useful to the research team! I'm not sure how it affected patient outcomes on my end. My patient was comfortable with being recorded and it didn't have a negative impact on the visit.  *Help patients receive proper care.* |  |
| RN5 | Forms | The equipment didn’t work. So I used my phone  *Positive experience.* | *Difficult* | It went well. I started at the beginning and ended the recording at the end. I didn’t interfere with the flow. Challenges is just the quiet moments when we talked about everything and Joe really knowing the recordings were loud enough.  *Challenge with microphone setup. Eliminated by using iPhone mic.* | I think they were more conscious of what they was saying and didn’t want to say the “wrong thing “  *Self-consciousness for patient.* | I think it’s a positive impact they were more intuned to what I was saying and was able to follow through and respond. They paid more attention  *Increases patient participation.* |  |
| PT4 | Zoom | Good experience. Received assistance for first visit and was able to easily perform recordings in subsequent visits.  *Positive experience.* | *Easy* | Very easy; no issues.  *No issues* | The knowledge of being recorded can cause self-consciousness for both clinicians and patients. It may make it difficult to discuss sensitive topics.  *Self-consciousness for both clinician and patient.*  *May limit communication of sensitive info.* | Recording patients’ speech can also help monitor breathing/shortness of breath as well as anxiety.  *Help patients receive proper care.*  *Improve communication of information.* | Some patients should have a more thorough evaluation for MCI.  May be beneficial for clinicians to talk with patients about study and provide their own feedback regarding patients’ eligiblilty. |
